# Harnessing fine fibers in decellularized adipose-derived matrix for enhanced adipose regeneration

**DOI:** 10.1016/j.mtbio.2024.100974

**Published:** 2024-01-25

**Authors:** Jiayi Feng, Su Fu, Jie Luan

**Affiliations:** Department of Aesthetic and Reconstructive Breast Surgery, Plastic Surgery Hospital, Chinese Academy of Medical Sciences and Peking Union Medical College, Beijing, 100144, China

**Keywords:** Decellularized adipose-derived matrix, Fiber, Structure, Adipose regeneration

## Abstract

Decellularized Adipose-Derived Matrix (DAM) has the function of inducing adipogenesis, but the distribution of adipogenesis is uneven. We found for the first time that DAM contains two structural components: The tough fibers DAM (T-DAM) and the fine fibers DAM (F-DAM). T-DAM was a dense vortex structure composed of a large number of coarse fibers, characterized by myoblast-related proteins, which cannot achieve fat regeneration and forms a typical "adipose-free zone". While the F-DAM was a loose structure consisting of uniform fine fibers, has more matrix-related proteins and adipose-related proteins. It can not only better promote the adhesion and proliferation of adipose stem cells in vitro, but also achieve the regeneration of adipose tissue *in vivo* earlier and better, with a uniform range of adipogenesis. The F-DAM is the main and effective kind of DAM to initiate adipose tissue regeneration, which can be picked out by ultrasound fragmentation.

## Highlights

• The decellularized adipose-derived matrix is not a uniform scaffold, and it is classified for the first time.
• DAM can be divided into tough fibers DAM (T-DAM) and fine fibers DAM (F-DAM).
• F-DAM is an effective DAM to initiate adipose tissue regeneration.

## Graphical abstract

**Figure:**
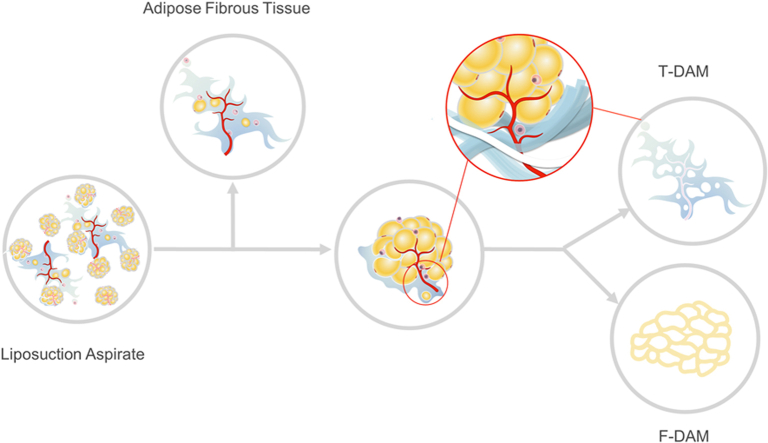

## 1 Introduction

At present, tissue engineering regeneration and repair reconstructions can be divided into the following three types: one is transplantation of other body parts into the target area [1,2], the other is the scaffold or patch alone or with cells, growth factors, or exosomes [[3], [4], [5]], and the third is regeneration *in situ* of scaffolds or analogs [1,6,7]. The first method is currently the most commonly used, while regeneration *in situ* has always been the ideal way to repair tissue defects. It does not require donor tissue and avoids additional trauma to the donor area. Moreover, there is no in vitro culture process, avoiding complex processes such as cell variation, contamination, complex cell culture and tissue establishment. It also avoids problems such as transplant trauma, immunogenicity, infection, pathogen transmission, and medical ethics. Since tissue and blood vessels regenerate simultaneously, there are no problems with revascularization. Finally, it requires no secondary transfer operation and is economical and convenient.

Decellularized Adipose-derived Matrix (DAM) is one of the few biological materials capable of inducing fat regeneration *in situ*. It does not require exogenous mesenchymal cells, but relies only on the recruitment of endogenous seed cells and the continuous induction of cell homing to the scaffold. DAM induces tissue regeneration rather than tissue repair, which means that it is eventually replaced by healthy, functional host adipose tissue [8,9].

In previous studies of adipose tissue regeneration induced by DAM, the effect of adipogenesis varied widely after transplantation *in vivo*. Even at the later stage of implantation, there is still uneven adipogenesis, meaning that adipogenesis does not occur in some areas [[10], [11], [12]]. This could be related to the different tissue sources or preparation methods of DAM. We found that in the DAM, which were prepared using conventional enzymatic methods, some swirling, dense fiber structures could be detected in the HE staining, which were clearly different from those of the loose fibers. These fibers, characterized by a spiral structure, are defined as "Tough DAM" (T-DAM), while the other, more delicate and loose fibers are defined as "Fine- DAM" (F-DAM).

In previous studies, DAM was considered to be a unitary acellular scaffold. However, we have found that the structure and composition of DAM is not uniform. We inferred that the reason for the uneven range of adipogenesis in DAM might be the different distribution and proportion of T-DAM and F-DAM. However, what the difference is between the composition and structure of T-DAM and that of F-DAM. And what the difference is in the effect of inducing fat regeneration *in vivo*. The answers to these questions will not only have a significant impact on the effect and efficiency of DAM inducing adipose tissue regeneration, but may also change the previous experimental conclusions and thus our understanding of DAM. To this end, the morphological structure, composition, and ability of adipogenesis in T-DAM and F-DAM were investigated.

## 2 Methods

### 2.1 Preparation of decellularized adipose-derived matrix

Adipose tissue was obtained from 10 healthy female patients, aged 37.27 ± 4.86 years with a BMI of 20.93 ± 1.38 kg/m^2^. The collection and use of adipose tissue was reviewed and approved by the Ethics Committee of the Plastic Surgery Hospital of the Chinese Academy of Medical Sciences (No. ZX201843).

Separation of tough and fine fibers: DAM preparation was performed according to our previous method [13]. Briefly, the coarse fibers were picked out and cleaned as much as possible with sharp forceps. Then, the adipose tissue was repeatedly crushed with ultrasonic cell crusher at 90W power under ice bath stirring. In this process, the tough fibers were aggregated into a group and easily separated. The remaining suspension was centrifuged at 4000 rpm for 3 min, and the fine fibers were obtained after the top layer of oil is discarded.

Decellularized treatment: The separated tough and fine fibers were soaked in 1 % Triton-X100 solution in a constant temperature shaker (37 °C, 100 rpm) for 48h, respectively. And then rinsed with sterile distilled water under the shaker at step intervals of 10min, 30min, 1h, 2h and overnight (>12h). After adding the isopropyl alcohol on shaker for 6h to remove remaining lipids. Rinse with sterile distilled water and 75 % alcohol 3 times for 30min each time. Finally, T-DAM and F-DAM were obtained and stored in 1 % penicillin-streptomycin solution at 4 °C.

### 2.2 Morphological and histological contrast

The morphology of adipose tissue, T-DAM and F-DAM were observed. Comparison of structure and composition was performed on DAMs from three batches (n = 3). The DAMs were fixed with 4 % paraformaldehyde and cut into 4 μm thick sections after paraffin embedding. Hematoxylin-eosin (HE) staining was performed according to standard procedures, including dewaxing, soaking, staining, differentiation, blue staining, dehydration, transparency, etc. For Sirius red staining, the paraffin slices after dewaxing were immersed in the Sirius red dye solution for 8 min after deparaffinization, and the images were taken under polarized light after dehydration and transparency. Type I collagen is visualized as an orange or red fiber, while type III collagen presented as a green one. For immunofluorescence staining, after dewaxing, antigen repair and sealing, the paraffin sections were incubated with anti-laminin (Abcam, Cambridge, UK) and the secondary antibody CoraLite594 (Proteintech, Wuhan, China). The results of fluorescence staining were observed after panoramic scanning using the Microdigital scanning system (Motic EasyScan Pro 6).

#### 2.2.1 Scanning electron microscopy

The DAMs were fixed with Gluta fixing solution (P1126, Solarbio, Beijing, China) at room temperature for 2h. After fixation, gradient dehydration and drying, gold spraying for 30s, the two DAMs were observed and photographed by scanning electron microscope (SU8100, HITACHI Ltd., Tokyo, Japan).

### 2.3 Glycosaminoglycan quantification

Glycosaminoglycan (GAG) is considered the most important active protein of DAM. Quantification was performed using the Blyscan GAG detection kit (Biocolor, United Kingdom). DAMs were freeze-dried and incubated with papain (25 mg/ml) at 60 °C for 3 h. Papain digested samples were analyzed according to the manufacturer's instructions (n = 3). Absorbance was measured at 650 nm, 3 compound holes were repeated for each sample, and GAG content was quantified using a standard curve.

### 2.4 Growth factor quantification

The concentrations of bFGF and EGF in both DAMs were measured using the Luminex® Multifactor Assay Kit (R&D Systems®, LXSAHM-04) (n = 3). After homogenization, total tissue protein was quantified and a standard curve was generated. According to the manufacturer's instructions, 50 μl of standard and a diluted protein sample were added to each test well, followed by 50 μl of diluted biotin-antibody mixture and 50 μl of diluted PE-labelled streptavidin. Incubation was performed on a microplate oscillator (IKA, Staufen, Germany) at room temperature for 1 h in the absence of light. After purification and suspension with the designated purification buffer, the assay was performed on a flow analyzer (LSRFortessa SORP, BD, NJ, USA) with 3 compound holes in each sample. After detection, the concentration of the growth factor was determined using the corresponding fluorescence value of the standard curve.

### 2.5 Biomechanical properties test

The mechanical properties of the two DAMs were measured with a biomechanical tester (Instron 5967, Norwood, MA). Using a corneal trephine, the F-DAM and T-DAM specimens were cut into cylindrical pieces 6 mm in diameter and approximately 2 mm thick (n = 3) and compressed at 10 mm/min using a 10 N load sensor. The Young's modulus was calculated from the slope of the initial linear portion of the stress-strain curve. The stress-strain curve was plotted using Origin9 after Green's strain transformation was applied.

### 2.6 Protein label-free quantification and bioinformatics analysis

The F-DAM and T-DAM were subjected to label-free quantification (LFQ) proteomic analysis. After quantification of total BCA protein in two groups of samples (n = 3), protein reduction, alkylation and digestion were performed to obtain peptide segments. Then, 1 μg of total peptide was collected from each sample and separated by high-performance liquid chromatography (nanoUPLC EASYnLC1200, Thermo Scientific, USA). Data acquisition was performed in conjunction with a mass spectrometer equipped with a nanoliter ion source (QE-xactive HFX, Thermo Scientific, USA). Proteome Discoverer software (version 2.4.0.305, Thermo Fisher Scientific) and Sequest HT search engine were used for database search analysis, and L-DAM and S-DAM differential proteins were obtained. The screening criteria for differentially expressed proteins were p-value ＜0.05 of Student's t-test or chi-squared test, with fold change≥1.2 [14]. Intensity-based absolute quantification (iBAQ) has been used to calculate the content of important proteins [15,16]. Gene Ontology (GO) annotation enrichment analysis, KEGG annotation enrichment analysis and gene set enrichment analysis (GSEA) were performed.

### 2.7 Co-culture of DAM with adipose-derived stem cells

Adipose-derived stem cells (ADSCs) were extracted from the fat aspirates of the above patients. ADSCs were isolated by 0.1 % type I collagenase at 37 °C for 45 min. They were cultured in Dulbecco's modified Eagle's medium (DMEM) containing 10 % fetal bovine serum (FBS). P3 of ADSCs were selected as the research object in this project. 5 mg T-DAM and 5 mg F-DAM was taken, respectively. The DAMs in vitro were all derived from three batches of DAMs (n = 3). After the DAMs were washed with sterile PBS, 10 μl of cell suspension containing 1 × 10^5^ ADSCs and DMEM were slowly added to each DAM. Subsequently, 490 μl DMEM was added to a 24-well plate for culture.

The next day, the recellularized DAMs were transferred to a new well plate for further culture.

### 2.8 Evaluation of cell adhesion and cell activity to DAMs

LIVE/DEAD® viability/cytotoxicity detection kit (Thermo Fisher) was used to observe cell adhesion of FDAM-ADSC group and TDAM-ADSC group on day 1 and day 7 after co-culture (n = 3). The confocal microscope (Leica, Allendale, N.J.) was used to capture images of the DAM scaffold for observation. The Image J software was used to calculate the number of adipocytes per field.

### 2.9 The ability to induce adipogenic differentiation in vitro

FDAM-ADSC group and TDAM-ADSC group (n = 3) were co-cultured in 24-well plates for 7 days each and then cultured with human adipogenic mesenchymal stem cells adipogenic induction differentiation kit (HUXMD-90031, Oricell, CN) for 2 weeks. At the same time, FDAM without transplanted cells was used as a blank control. The ADSCs-DAM complex was fixed with 4 % paraformaldehyde, stained with oil red O solution, added with 100 % isopropyl alcohol solution, incubated at room temperature for 20 min, and the oil red in the complex was eluted into isopropyl alcohol. After the elution was uniformly blown, 100 μL eluent was absorbed and added to the 96-well plate. The OD value was measured at a wavelength of 520 nm using a microplate reader and zeroed by the blank control group.

Lipid formation of the FDAM-ADSC group and the TDAM-ADSC group was observed by Bodipy and Hoechst fluorescence staining. Prepare a working solution of Hoechst 33342+Bodipy (Thermo, United States) according to the kit protocol, shake in the dark for 30 min, and rinse twice with PBS. The confocal microscope (Leica, Allendale, N.J.) was used to capture images for observation. The Image J software was used to calculate the number of adipocytes per field.

### 2.10 Animal experiment *in vivo* of adipogenesis on DAMs

Balb/c nude male mice aged 6–8 weeks were used in this experiment (Huafukang, Beijing, China). The study was approved by the Ethics Committee of the Plastic Surgery Hospital of the Chinese Academy of Medical Sciences [2023(2)]. Animal experimentation procedures strictly followed the regulation and standards for the protection and use of laboratory animals formulated by the Chinese Academy of Medical Sciences and Peking Union Medical College.

T-DAM and F-DAM 50 μl were thoroughly cut, mixed with normal saline, and injected into the left and right subcutaneous back of mice using an 18-gauge needle. Mice were euthanized at 1, 4, and 8 weeks after injection (n = 5 at each time point).

### 2.11 Animal experiment *in vivo* of adipogenesis between powdered T-DAM and bulk T-DAM

Considering the swirling scaffold structure in T-DAM may be the reason that makes it difficult to achieve adipogenesis. The T-DAM is lyophilized using vacuum freeze-dryer. Part of the T-DAM is cut with scissors into about 1mm3 particles, which is called bulk T-DAM (B-TDAM). The other part was ground into powder using a tissue grinder (Tissuelyser-24L, Jingxin Ltd., Shanghai, China) after freezing with liquid nitrogen. It was found that there was no obvious spiral structure in HE staining, which was named powder L-DAM (P-TDAM). Both T-DAMs were sterilized with cobalt-60 radiation before use. B-TDAM and P-TDAM 5 mg were fully mixed with normal saline and injected into the left and right back of mice, respectively. Implant samples were collected by euthanizing mice 4 weeks after injection (n = 5).

### 2.12 Histological and immunohistochemical staining

After the specimens were fixed in 4 % paraformaldehyde for 24–48 h, the tissues were cut at the center of the longest diameter, embedded in paraffin and sectioned. HE was used to evaluate the morphology of the implants at each time point. The lipogenic marker perilipin-1 (Abcam, Cambridge, UK) was stained by immunohistochemistry. CD31 (Abcam, Cambridge, UK) was used to localize vascular endothelial cells. Panoramic scanning of sections was performed with Microdigital section scanning system (Motic EasyScan Pro 6). Image J software was used to calculate the area percentage of panoramic perilipin-1-positive adipocytes to assess in-implant adipocyte regeneration. Adipocytes were randomly selected to measure cell diameter (the longest diameter was calculated for non-circular adipocytes). The area percentage of CD31-positive vessels at 100x was calculated to assess in-implant angiogenesis.

### 2.13 Statistical analysis

All data are expressed as mean ± standard deviation (SD). Statistical analysis was performed using Prism 9.0 software (Graphpad Software, USA). The independent samples *t*-test was used to evaluate the physical properties of the two DAMS. And the statistical significance of data collected over multiple time periods *in vivo* was analyzed at the 95 % confidence level using analysis of variance. p < 0.05 is considered statistically significant (*p < 0.05，**p < 0.01，***p < 0.001).

## 3 Results

### 3.1 Characteristics of T-DAM and F-DAM

**Fig. 1:**
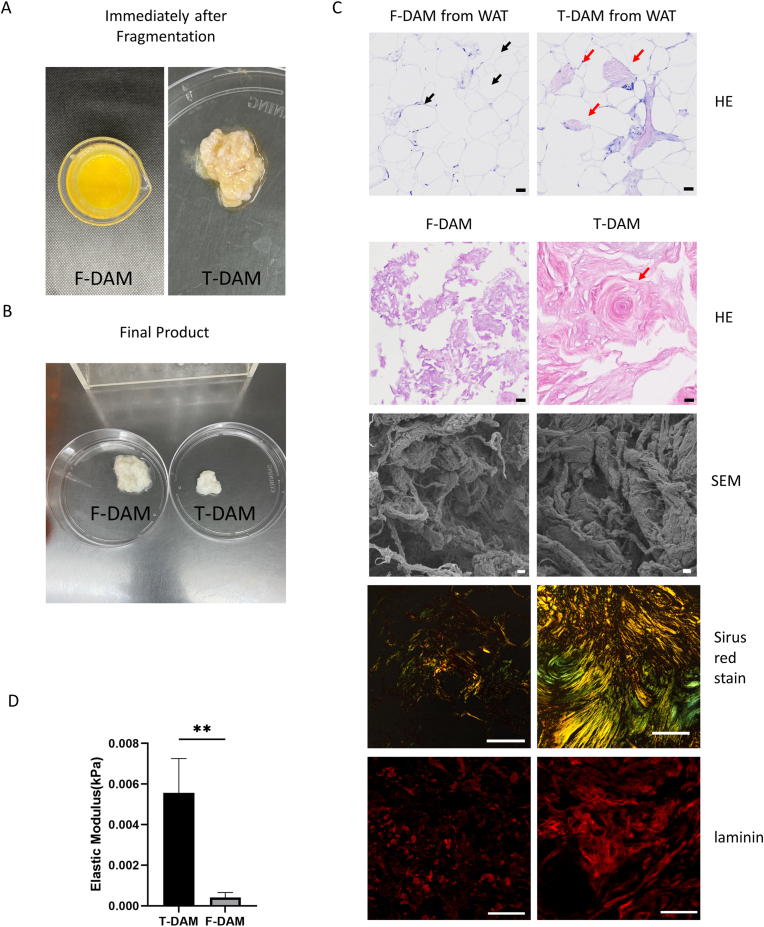
Macroscopic and microscopic characteristics of T-DAM and F-DAM. (A) The initial of T-DAM and F-DAM immediately broken and separated after ultrasonication. (B)General appearance comparison of T-DAM and F-DAM. Biomechanical curves of T-DAM and F-DAM. (C) HE staining of adipose tissue, red arrow for T-DAM, black arrow for F-DAM (Scale bar = 30 μm), SEM staining (Scale bar = 10 μm), Sirius red staining (Scale bar = 100 μm) and laminin immunofluorescence staining (Scale bar = 100 μm). (D)Elastic modulus of T-DAM and F-DAM.. (n = 3, each group of samples derived from three batches of DAMs.). (For interpretation of the references to color in this figure legend, the reader is referred to the Web version of this article.)

After the adipose tissue was crushed by ultrasound (Fig. 1A), the initial DAM products were divided into two types: T-DAM (fibrous tissue) and F-DAM (suspension). Then, the acellular operation was continued by the enzyme-free method. DAM, which were prepared by different individual and liposuction methods, vary greatly in the ratio of T-DAM and F-DAM, ranging from 9:1–3:2. Both T-DAM and F-DAM are white solids when viewed in general (Fig. 1B). In contrast, T-DAM is white in color and its tissue contains a hard, crimped and thickened part. Most of F-DAM is soft and yellowish flocculent. In HE staining, the corresponding structures of both DAMs can also be found in human white adipose tissue (Fig. 1C). The F-DAM is a uniform fine fibrous structure, while the T-DAM contains a large number of vortex-like dense fibrous structures. In the scanning electron microscope, the structure of DAMs consists of small fibers that are intertwined and wound into large fiber clusters. It can be seen that the F-DAM has smaller fiber clusters and looser fiber structure, while the F-DAM has larger fiber clusters and more dense structure.

Both Sirius red and fluorescence staining showed that T-DAM contained more type I and type III collagen and laminin. However, there was no significant difference in GAG content between T-DAM and F-DAM (p = 0.777), which was 0.98 ± 0.74 μg/mg and 0.84 ± 0.24 μg/mg, respectively. Although the levels of EGF and bFGF in F-DAM were significantly higher than in T-DAM, the levels were also very low. The EGF content of T-DAM and F-DAM was 0.07 ± 0.02 μg/mg and 0.14 ± 0.01 μg/mg, respectively (p = 0.009), and the bFGF content was 0.04 ± 0.02 μg/mg and 0.10 ± 0.01 μg/mg, respectively (p = 0.007).

The mechanical characteristics of the two DAMs were evaluated (Fig. 1D). It is found that the elastic modulus of F-DAM is obviously smaller than that of T-DAM (p = 0.006).

### 3.2 Comparison of label-free quantification proteomic analysis

**Fig. 2:**
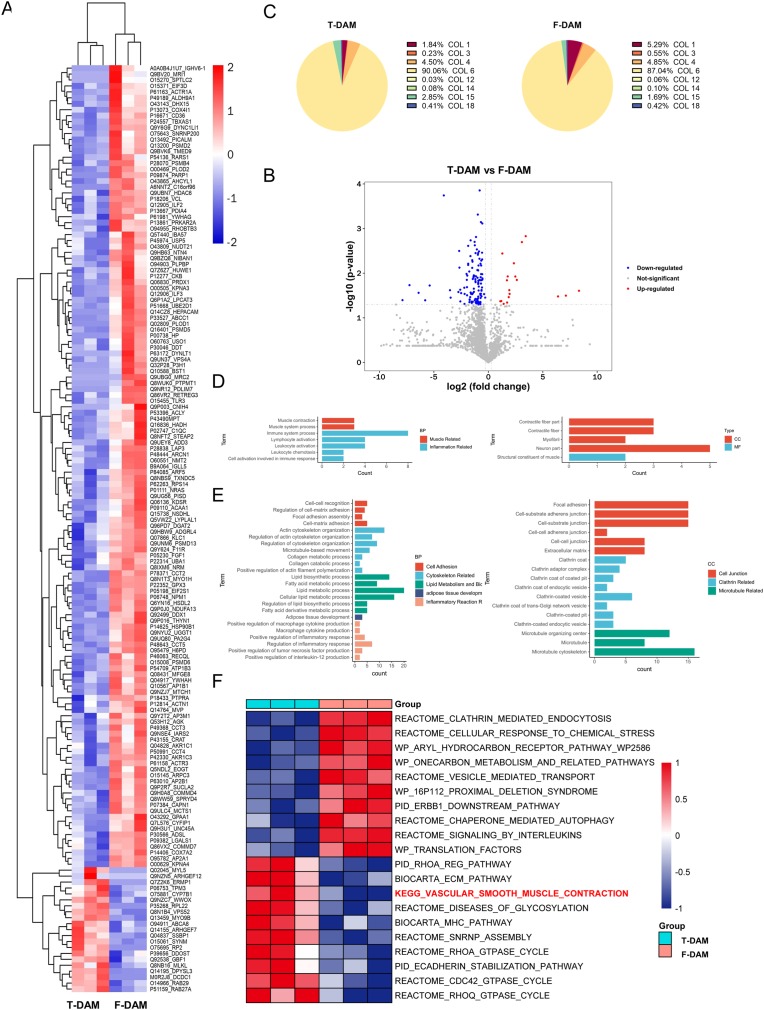
LFQ analysis of T-DAM and F-DAM (A) Hierarchical cluster analysis heat map of T-DAM and F-DAM (B) Collagen types and content of T-DAM and F-DAM. (C) Volcano map of differentially expressed protein quantity of T-DAM and F-DAM. (D) Critical differential GO pathway enrichment analysis of T-DAM. (E) Enrichment analysis of GO differential pathway of F-DAM. (F) Gene set enrichment analysis (GSEA) for T-DAM.

To understand the difference in total protein composition between T-DAM and F-DAM, label-free quantification (LFQ) was performed. The heat map shown the difference between the proteomic profiles of T-DAM and F-DAM (Fig. 2A). A total of 2434 proteins were detected, of which 156 were significantly different, as shown in the **Supplementary 1**. 135 proteins were significantly upregulated in F-DAM, whereas only 21 were significantly upregulated in T-DAM (Fig. 2B). Based on the Matrisome Project (http://Matrisome project.mit.edu/), the matrisome-related proteins that were significantly different between the two DAMs were excavated. Some upregulated matrix proteins were found in F-DAM, as shown in Table 1. No significantly upregulated matrix-related proteins were found in T-DAM. Among the upregulated matrix proteins, Netrin 4 (NTN4) plays a crucial role in vascular development by binding to laminin 1, to promote cell migration and tissue development [17]. Galectin 1 (LGALS1), as a laminin binding protein, participates in cell attachment, migration, growth, and forms a network with type IV collagen α chain [18,19]. Fibroblast Growth Factor 1 (FGF1) is recognized for regulating cell survival, division, differentiation, and migration while effectively inducing angiogenesis [20]. Zhang et al. [21] have demonstrated the higher efficiency of DAM loaded with bFGF in promoting adipose neotissue formation and neovascularization. In the Procollagen‐Lysine, 2‐Oxoglutarate 5‐Dioxygenase (PLOD) family, PLOD1 and PLOD2 are known to be essential for intermolecular collagen crosslinking stability necessary, as well as normal assembly and crosslinking of collagen fibrils, associated with tissue development [22]. However, there were no significant differences in the type and content of collagen in either T-DAM or F-DAM. The most abundant collagen was type VI, followed by type I and type IV (Fig. 2C). Details of the content of each subtype and the number of unique peptides were provided in **Supplementary 2**.

**Table 1:** Matrisome-related proteins in F-DAM.

Function	Protein	P Value
ECM Glycoproteins	NTN4	0.017
ECM-affiliated Protein	C1QC LGALS1	0.041 0.020
ECM Secreted Factors	FGF1	0.021
ECM Regulators	PLOD1 PLOD2	0.028 0.006

At the same time, based on the gene ontology (GO), we also found the difference in protein function between T-DAM and F-DAM. GO annotation is used to classify cells according to three categories: cell composition (CC), molecular function (MF) and biological process (BP). We classify the distinct GO pathways specifically for DAMs. In BP (Fig. 2D and E), the specific pathways in F-DAM include aspects such as cell adhesion, cytoskeletal related, collagen synthesis and degradation, and lipid metabolism and biosynthesis. Among them, the pathway of “adipose tissue development” (GO:0060612) is only significantly enriched in F-DAM, but not found in T-DAM. The specific pathways of T-DAM are related to muscle structure and neurons. In CC, both DAMs are located in "extracellular organelles"(GO:0043230), "extracellular space"(GO:0005615), "extracellular exosomes"(GO:0070062), "extracellular region portion” (GO:0044421), and only fine fibers are located in the "extracellular matrix"(GO:0031012). Enrichment of Clathrin protein family, laminin and other specific pathways related to ECM remodeling was also found in F-DAM.

In addition, in order to explore the differences in the biological functions of these two DAMs, we also performed GSVA enrichment analysis. In GSVA analysis (Fig. 2F), F-DAM were significantly enriched in the "vascular smooth muscle contraction" pathway, while it was not found in T-DAM.

### 3.3 Differences in adhesion and proliferation of adipose stem cells on DAMs and adipogenic differentiation in vitro

**Fig. 3:**
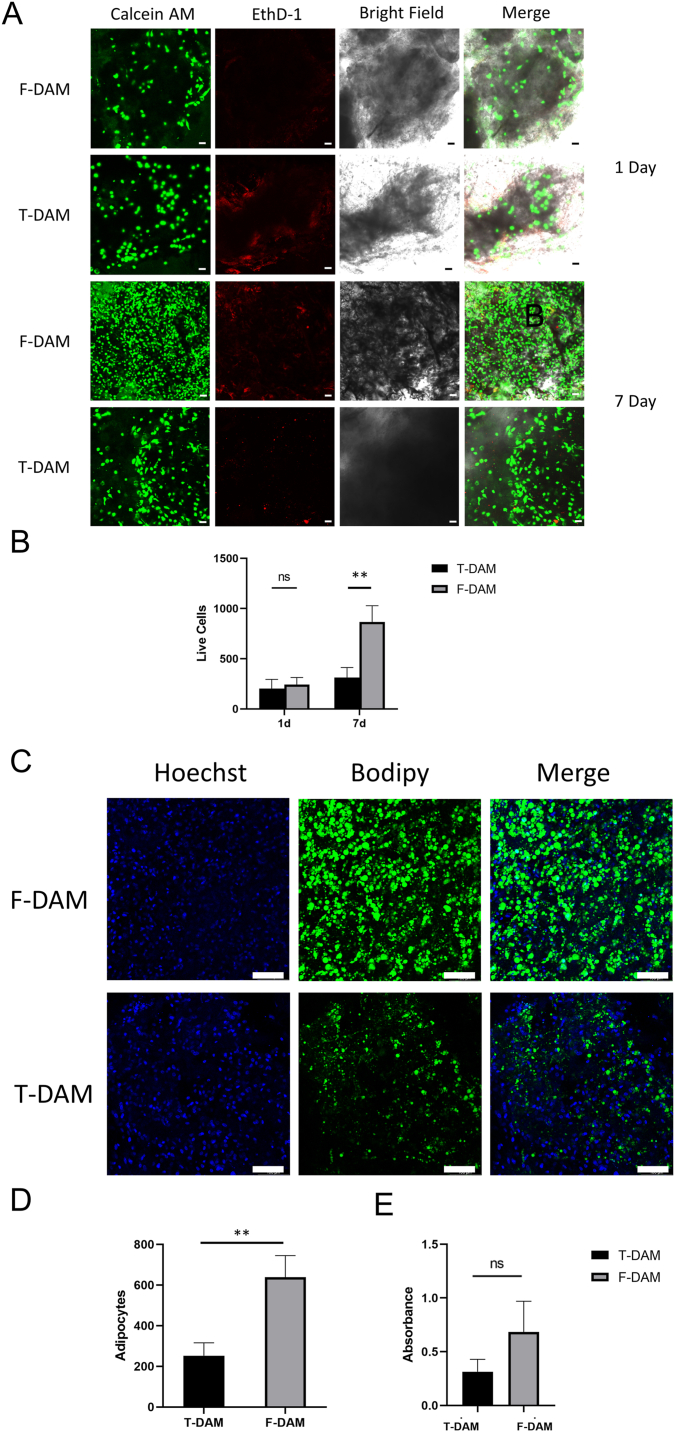
In vitro co-culture of ADSCs with DAMs. (A) Cytotoxic live and dead staining of ADSCs co-culture with DAMs. Scale bar = 100 μm. (B) Cell adhesion and proliferation of ADSCs co-culture with DAMs. (C) Bodipy fluorescence staining in lipogenesis induced of ASCs co-culture with DAMs. Scale bar = 100 μm. (D) The number of adipocytes per field of lipogenesis induction after co-culture of ADSCs with DAMs. (E) Comparison of absorbance of lipogenesis induced by oil red staining after co-culture of ADSCs with DAMs. (For interpretation of the references to color in this figure legend, the reader is referred to the Web version of this article.)

On the first and seventh days of culture, cell adhesion and proliferation of ADSCs of the two DAMs were assessed (Fig. 3A). Calcein AM indicates that live cells are stained green, whereas ethidium homodimer-1 (EthD-1) indicates that dead cells are stained red. We found that both DAMs attached to a similar number of ASCs at 1d (p = 0.575). However, the proliferation of ASCs on F-DAM was much greater at day 7(p = 0.007), although the number of dead cells was small and similar between the two groups (Fig. 3B). T-DAM and F-DAM were each inoculated with ADSCs, underwent adipogenic differentiation. As can be seen from Fig. 3C, there were significantly more green droplets stained by Bodipy in F-DAM (p = 0.006) (Fig. 3D), but no statistical difference was detected in oil red staining(p = 0.106) (Fig. 3E).

### 3.4 DAMs induced adipose regeneration *in vivo*

**Fig. 4:**
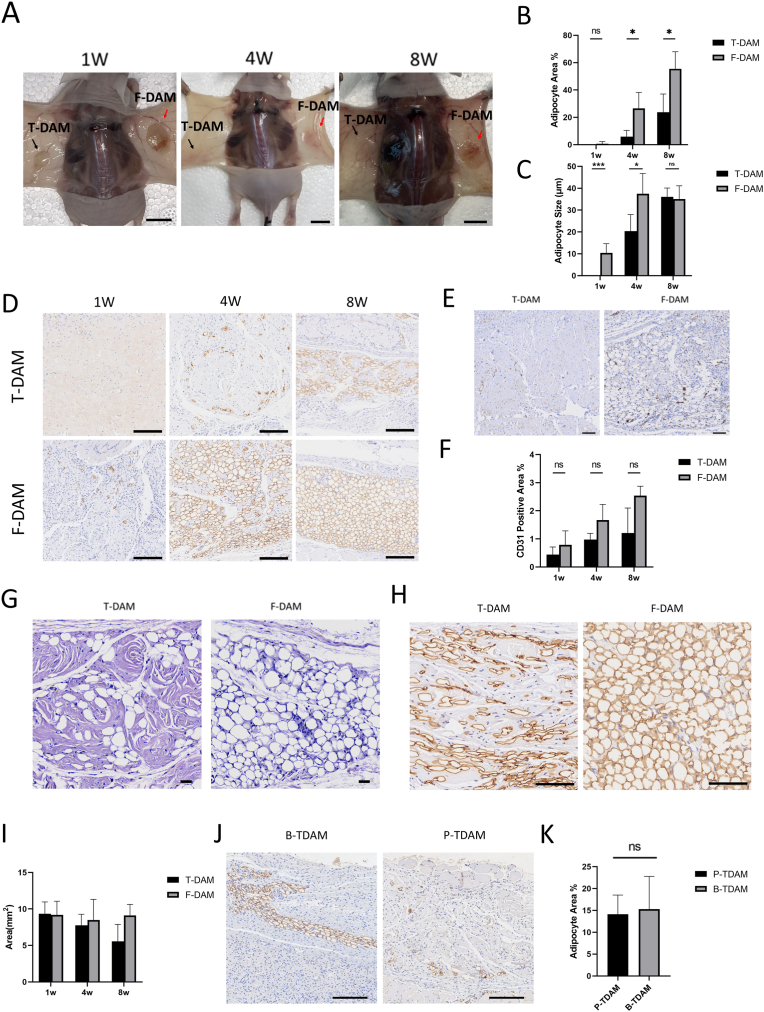
The regeneration of adipose tissue after the injection of T-DAM and F-DAM. (A) Gross observation after the of T-DAM and 8w F-DAM. Scale bar = 1 cm. (B) Adipocyte area ratio after T-DAM and F-DAM implantation. (C) Comparison of adipocyte diameters in different periods. (D)Perilipin-1 staining of adipocytes after T-DAM and F-DAM implantation. Scale bar = 200 μm. (E) CD31 staining after T-DAM and F-DAM implantation at 4w. Scale bar = 100 μm. (F)Vascular area ratio after T-DAM and F-DAM implantation. (G) HE staining of adipocytes after T-DAM and F-DAM implantation at 8w. Scale bar = 30 μm. (H) Perilipin-1 staining of adipocytes after T-DAM and F-DAM implantation at 8w. Scale bar = 100 μm. (I) Comparison of the maximum cross section of implants of T-DAM and F-DAM in different periods. (J) Perilipin-1 staining of adipocytes after B-TDAM and P-TDAM implantation at 4w. Scale bar = 200 μm. (K) Adipocyte area ratio after B-TDAM and P-TDAM implantation at 4w.

The nude mice were injected with T-DAM on the left side of the back and F-DAM on the right side. As can be seen from the general observation of the bilateral injected objects (Fig. 4A). At the first week, the injected objects on both sides were similar, round, white soft tissues, with F-DAM appearing darker red as a whole and having distinct vessels on its surface. After 4 weeks, the soft tissue boundaries on both sides were slightly blurred, T-DAM was still generally white, while F-DAM was pink overall and a large number of microvessels were visible to the naked eye. After 8 weeks, T-DAM had become a translucent white tissue block, the border was no longer clearly visible, and the volume had a marked tendency to shrink. F-DAM, on the other hand, showed stable volume, a clear tissue boundary, and a significantly larger vessel diameter with a large number of microvessel branches. CD31 staining also confirmed that the vessel diameter of F-DAM was significantly larger than that of T-DAM at 8w (T-DAM vessel diameter = 18.96 ± 5.74 μm, F-DAM vessel diameter = 66.90 ± 11.73 μm, p < 0.001).

Immunohistochemical staining of perilipin-1 allowed a more accurate assessment of adipocyte growth at the histological level after injection of the two DAMs (Fig. 4B CD). In the first week, new adipocytes (2 of 5) were observed in the F-DAM group, most of which were multilocular and had a small diameter of 10.42 ± 3.79 μm, whereas no newborn adipocytes were observed in the T-DAM group (5 of 5). A large number of mature adipocytes with a diameter of 39.42 ± 8.38 μm were observed at week 4 with F-DAM. However, the morphology of adipocytes in the T-DAM group at week 4 was mostly similar to that of naive adipocytes in the first week of the T-DAM group, which was 16.93 ± 7.59 μm. The regenerated adipocytes in the T-DAM group were separated by a large number of spiral fiber structures, were very sparsely distributed, and all grew around the periphery of the spiral fiber structure, showing a "flower ring" growth pathway. The proportion of adipocytes in T-DAM was much lower than that in F-DAM (p = 0.039). At week 8 after implantation, adipocytes in group F-DAM were almost complete, and growth was dense and uniform. The diameter of adipocytes slightly decreased to 31.20 ± 5.84 μm compared with the 4th week. The shape of the cells changed from quasi-circular to almost perfectly circular. Nevertheless, the morphology of F-DAM cells was strongly altered. Although the adipocytes were mainly circular, some local cells were crescent-shaped (Fig. 4H), with a length-diameter of 34.31 ± 7.61 μm. The percentage of adipocytes in the T-DAM group was still significantly lower than in the F-DAM group (p = 0.014). In terms of vascularization (Fig. 4E), there was no significant difference in the degree of vascularization between T-DAM and F-DAM at weeks 1, 4 and 8 (Fig. 4F) (1w p = 0.523, 4w p = 0.138, 8w p = 0.074). The HE staining of the two groups 2 months after injection (Fig. 4G) showed that a large number of helical dense structures, and thus a large number of helical lipid-free regions were still present in the T-DAM group, whereas no similar helical structures were significantly found in F-DAM. As for the retentional volume of implants (Fig. 4I), there was no significant difference in maximum cross-sectional area between T-DAM and F-DAM at 8w (p = 0.054).

Four weeks after implantation of P-TDAM and B-TDAM *in vivo*, perilipin A staining (Fig. 4J) showed no significant improvement in lipogenesis induced by the two methods (Fig. 4K). (p = 0.766).

## 4 Discussion

DAM is a potential biological scaffold in regeneration engineering. Regulation of adipogenesis *in situ* is the focus of research [8,23]. Because there are many DAM production methods, the remaining key components of DAM, obtained by different methods, are not identical. Due to the different delivery forms of the finished products, their internal structure is different, making it difficult to make a horizontal comparison of their ability to form lipids.

In addition to the fat particles themselves, adipose tissue consists of three different types of tissue: connective tissue, muscle tissue (such as the smooth muscle in the veins and arteries within the adipose tissue), and nervous tissue (such as the sympathetic nerves associated with adipose tissue storage and breakdown) [24,25]. Esteve et al. ^26^found that the interlobular ECM in adipose tissue was composed of two ECM compartments with different structures: the stroma ECM and the septa ECM. The fibers of the stroma ECM are small and sparse, and MSCA1+/CD271-progenitor cells with a high tendency to form lipids are enriched in their vicinity. The septa ECM fibers have a dense structure and are enriched with progenitor cell subsets of myoblasts with MSCA1-/CD271+, which have a higher intrinsic capacity to form myoblasts, which is related to the formation of a fibrotic diaphragm. In our study, we also found that DAM contains two different types of DAM, T-DAM and F-DAM. Based on the fact that adipose tissue contains two types of ECM with different adipogenic inducibility and differentiation tendency, we speculate that the T-DAM, formed by ECM with dense helical structure, may be similar to septa ECM, and its structure and induced microenvironment are not conducive to lipid formation. Therefore, F-DAM, which is similar to stroma ECM, is really conducive to adipogenesis. T-DAM and F-DAM originate from the different composition structure of adipose tissue, which is the main reason for their differences.

Based on the results observed under the type microscope and electron microscope, we believe that the T-DAM consists of the thickened fiber components in adipocytes, such as the septa ECM and the vascular smooth muscle in adipocytes, which is characterized by the dense spiral structure under the microscope. The F-DAM is the stroma ECM inside the fat lobule, and its fibers are sparse and thin, which is the ECM source for lipid differentiation. From the analysis of protein components, the distinguishable components of F-DAM are matrix-related proteins, adipogenesis-related proteins, and ECM remodeling-related proteins, whereas T-DAM is particularly rich in muscle-related proteins. In terms of mechanical structure, the elastic modulus of F-DAM is significantly lower than that of T-DAM, suggesting that the stiffness is also lower. A harder matrix may promote osteogenic differentiation rather than lipogenic differentiation of ASCs [[27], [28], [29]]. All this suggests that F-DAM is the natural microenvironment for adipose tissue regeneration, whereas T-DAM is not an ideal scaffold for adipocyte settlement, differentiation and proliferation.

In addition, the lipid-forming ability around F-DAM was significantly better than that around T-DAM, although there was no significant difference in vasoforming ability between the two, indicating that the poor adipogenesis of T-DAM was not related to the degree of vasogenesis. At the same time, our experiment also showed that the environment of T-DAM was not completely incapable of adipocyte formation. We hypothesized that this was mainly related to the inevitable attachment of a small amount of F-DAM to the T-DAM. After all, the DAM exists in a flocculent form that is easily wound. Considering the characteristic spiral structure of T-DAM, in order to exclude that it is the dense structure that leads to the difference in fat formation of DAM, we designed an experiment to observe whether the fat-forming ability of T-DAM is significantly improved after completely crushing. The final results showed that the lipid forming potential was not significantly increased even after grinding. This proves that the structure of T-DAM is not the only factor limiting its adipogenic ability.

Adipogenesis is a continuous cellular process that begins with ADSCs, mesenchymal stem cells (MSCs), or other pluripotent stem cells [[30], [31], [32]]. It has now been widely demonstrated that the adipogenic capacity varies depending on the fat depot [[33], [34], [35], [36]], which may be caused by the types of seed cells attracted, the chemokines and associated growth factors. Ultimately, however, it can be attributed to differences in the tissue microenvironment. It has differentiation selectivity under the influence of different ECM niches. The stromal ECM or F-DAM niche, which is conducive to lipid formation, is dedicated to differentiation into lipid lineages. However, in the microenvironment of septa ECM and T-DAM, a significant number of progenitor cells may choose to differentiate in other directions, such as myofibrosis, rather than adipogenesis. At the same time, it has been suggested that the loose structural composition of the ECM allows adipocyte hypertrophy. The physical constraints associated with the dense fiber septum would inhibit lobular expansion and adipocyte hypertrophy [26], and the same can be applied to T-DAM. Its helical, dense structure not only makes it difficult for seed cells to colonize, but also inhibits the ability of regenerative adipocytes to expand. Poor adipogenic potential limits both adipocyte proliferation and mature adipocyte renewal, resulting in the accumulation of dysfunctional mature adipocytes [26,37,38]. Therefore, even though F-DAM may have a small amount of lipid formation, the adipocytes around it are mostly deformed and atrophic, indicating its maturity and poor metabolism.

In our previous study, we have concluded that the adipogenesis ability of DAM produced by ultrasonic crushing is much better than that produced by other mechanical crushing by homogenizer or freeze ball milling. The intrinsic reason is that the homogenizer or other grinder uses high-speed cutting to break all thick or fine fibers as well as cells without any difference. The coarse fibers are crushed and thus mixed with the fine fibers. Coleman's classic fat grafting method also suggests picking out the visible fibers from the liposuction during the cleaning process [39,40]. During the pretreatment of adipose tissue, we deliberately picked out these large visible fibers from adipose tissue obtained by liposuction, which have been proved to be detrimental to adipocyte survival after fat grafting [41]. The cells contained in adipose tissue are complex, and it is difficult to completely isolate and eliminate nonadipose cellular components such as blood vessel components [42].

It is not possible to distinguish coarse fibers from fine fibers in adipose tissue unless ultrasonic crushing is performed. Ultrasound acts mainly on cells and cannot directly break ECM fibers at a certain power. The cavitation of ultrasound loosens the fiber gap and can be easily opened. As the exposure time of ultrasound increases, the coarse fibers and fine fibers are separated and exposed more thoroughly [[43], [44], [45]]. The self-assembly properties of collagen in vitro polymerize the separated coarse fibers into clusters [46], while the fine fibers are reversibly disassembled into smaller fibers and mixed in suspension, distinguishing the T-DAM and F-DAM. Previous studies have demonstrated that fibrillar collagen is not denatured by timely ultrasound exposure and its natural three-chain helical structure can be well preserved [47,48]. When the attached cells are broken up, the thickened coarse fibers lose the force of the cells on scaffold spreading, and become more wrinkled and curl up into a ball that is easily visible to the naked eye.

The septa ECM and stroma ECM can be accurately separated out at 80 × magnification under a type microscope [26]. However, in the preparation of DAM, we cannot separate the F-DAM and T-DAM before mechanical precrushing. First, a lot of adipose tissue is required for the preparation of DAM, and the yield of DAM is about 1 %. Second, the T-DAM, like the smooth muscle component of blood vessels, penetrates deep into the fat lobules and adjoins the F-DAM, so it cannot be excised. Moreover, DAM derived from human with different BMI can maybe lead to varying ratios of tough fibers and fine fibers. Therefore, pre-cutting with ultrasound is a necessary step to obtain F-DAM.

Therefore, in this experiment, we have proved that T-DAM is completely different from F-DAM by macro, micro, protein composition, structure and adipogenesis *in vivo* and in vitro. Only F-DAM is highly effective in inducing adipose tissue regeneration *in situ*.

## 5 Conclusion

In this study, we found for the first time that DAM consists of two structural components: the T-DAM and the F-DAM. The F-DAM has a better ability to induce adipose tissue regeneration *in vivo*. It is proved to be the main and effective structural component of DAM that can effectively induce adipose tissue regeneration. It can be separated and obtained by a simple ultrasonication.

## CRediT authorship contribution statement

**Jiayi Feng:** Writing - review & editing, Writing - original draft, Visualization, Validation, Supervision, Software, Resources, Project administration, Methodology, Investigation, Funding acquisition, Formal analysis, Data curation, Conceptualization. **Su Fu:** Writing - review & editing, Supervision, Project administration, Methodology, Conceptualization. **Jie Luan:** Writing - review & editing, Validation, Supervision, Resources, Project administration, Funding acquisition, Conceptualization.

## Declaration of competing interest

The authors declare that they have no known competing financial interests or personal relationships that could have appeared to influence the work reported in this paper.

## Data Availability

Data will be made available on request.
